# Novel Quantitative Analyses of Spontaneous Synaptic Events in Cortical Pyramidal Cells Reveal Subtle Parvalbumin-Expressing Interneuron Dysfunction in a Knock-In Mouse Model of Alzheimer’s Disease

**DOI:** 10.1523/ENEURO.0059-18.2018

**Published:** 2018-08-13

**Authors:** Lingxuan Chen, Takashi Saito, Takaomi C. Saido, Istvan Mody

**Affiliations:** 1 Molecular, Cellular, and Integrative Physiology Interdepartmental Ph.D. Program, The David Geffen School of Medicine at University of California, Los Angeles, Los Angeles, CA 90095; 2 Department of Neurology, the David Geffen School of Medicine at University of California, Los Angeles, Los Angeles, CA 90095; 3 Laboratory for Proteolytic Neuroscience, RIKEN Brain Science Institute, Wako, Saitama 351-0198, Japan; 4 Department of Neuroscience and Pathology, Research Institute of Environmental Medicine, Nagoya University 464-01, Japan

**Keywords:** Alzheimer’s disease, App knock-in mouse, excitation, inhibition, interneurons, parvalbumin

## Abstract

Alzheimer’s disease (AD) is a neurodegenerative disorder that has become a compelling global public health concern. Besides pathological hallmarks such as extracellular amyloid plaques, intracellular neurofibrillary tangles (NFTs), and loss of neurons and synapses, clinical reports have shown that epileptiform activity, even seizures, can occur early in the disease. Aberrant synaptic and network activities as well as epileptiform discharges have also been observed in various mouse models of AD. The new *App^NL-F^* mouse model is generated by a gene knock-in approach and there are limited studies on basic synaptic properties in *App^NL-F^* mice. Therefore, we applied quantitative methods to analyze spontaneous excitatory and inhibitory synaptic events in parietal cortex layer 2/3 pyramidal cells. First, by an objective amplitude distribution analysis, we found decreased amplitudes of spontaneous IPSCs (sIPSCs) in aged *App^NL-F^* mice caused by a reduction in the amplitudes of the large sIPSCs with fast rates of rise, consistent with deficits in the function of parvalbumin-expressing interneurons (PV INs). Second, we calculated the burstiness and memory in a series of successive synaptic events. Lastly, by using a novel approach to determine the excitation-to-inhibition (E/I) ratio, we found no changes in the *App^NL-F^* mice, indicating that homeostatic mechanisms may have maintained the overall balance of excitation and inhibition in spite of a mildly impaired PV IN function.

## Significance Statement

Using novel quantitative analyses of spontaneous synaptic currents in L2/3 pyramidal cells, we revealed subtle deficits in the function of parvalbumin-expressing interneurons (PV INs) in a new mouse model of Alzheimer’s disease (AD), the *App^NL-F^* mice. We applied novel statistical and analytical methods to further characterize the properties of spontaneous postsynaptic currents (sPSCs). Our approach provides rigorous quantitative tools for the analysis of synaptic events in mouse models of disease for objectively and reliably revealing even subtle disease-related alterations.

## Introduction

Alzheimer’s disease (AD) is a neurodegenerative disease that has become a global public health concern with the gradually aging population (Ferri et al., 2005; Mayeux and Stern, 2012). The pathologic hallmarks of AD include amyloid β (Aβ) plaques, neurofibrillary tangles (NFTs), and neuronal degeneration (Serrano-Pozo et al., 2011). Meanwhile, neuronal hyperactivity, network hyperexcitability, and spontaneous epileptiform activity have been observed in human amyloid precursor protein (hAPP) transgenic mouse models with high levels of Aβ (Palop et al., 2007, 2011; Busche et al., 2008; Palop and Mucke, 2009; Harris et al., 2010; Roberson et al., 2011; Yan et al., 2012; Busche and Konnerth, 2016). Hypersynchronous network activity, also observed in AD patients (Lam et al., 2017), may further contribute to the emergence of AD symptoms such as cognitive impairment in early stages of the disease (Noebels, 2011; Bakker et al., 2012; Scharfman, 2012). Specifically, electrophysiological studies in hAPPJ20 mice have revealed decreased intrinsic excitability of GABAergic parvalbumin-expressing interneurons (PV INs), which then led to aberrant network activity and cognitive deficits in the animals (Verret et al., 2012).

A key molecule in AD is Aβ, which is a 40 or 42 amino acid peptide derived from APP. Most of the studies on pathologic Aβ and subsequent alterations in synaptic and network activities have been using transgenic mouse models that overproduce mutant hAPP. Although these mouse models simulate several key aspects of human AD (Games et al., 1995; Hsiao et al., 1996; Sturchler-Pierrat et al., 1997; Götz et al., 2004; Cheng et al., 2007; Cissé et al., 2011; Verret et al., 2012), in these animals, Aβ and other APP fragments maybe overproduced and/or ectopically expressed (Chang and Suh, 2005; Mitani et al., 2012; Nicolas and Hassan, 2014; Kerridge et al., 2015; Nhan et al., 2015; Willem et al., 2015; Xia et al., 2016; Sasaguri et al., 2017). The *App^NL-F^* mouse model of AD was generated by manipulating the mouse *App* gene using a knock-in strategy (Nilsson et al., 2014; Saito et al., 2014), and this new mouse model is expected to advance our understanding of AD pathology. The paucity of studies characterizing the electrophysiological phenotype, such as synaptic transmission, in this model, prompted us to develop reliable and objective analytical approaches to identify possible synaptic changes in *App^NL-F^* mice.

Here, we have studied synaptic transmission in parietal cortex layer 2/3 pyramidal cells in the *App^NL-F^* mouse model of AD. The results showed a significant reduction in the average amplitude and average rate of rise (RR) of spontaneous IPSCs (sIPSCs) in old homozygous *App^NL-F^* mice compared to age-matched controls. Moreover, through amplitude distribution analysis, we found that the changes in the amplitude and RR were confined to fast-rising large amplitude sIPSCs but not the small ones, indicating potential impairment in perisomatic-targeting PV INs which has been described in the hAPPJ20 mice (Verret et al., 2012). We also used novel quantitative approaches to further examine the properties of spontaneous postsynaptic currents (sPSCs). Firstly, we applied methods previously used in characterizing the bursty nature of various successive events (Goh and Barabási, 2008; Schleiss et al., 2016) to evaluate the memory and burstiness of sPSCs, thus adding a new angle to the analysis of synaptic events. Secondly, we have developed a novel approach to calculate the excitation-to-inhibition (E/I) ratio with higher accuracy that incorporates all properties of sPSCs. Taken together, the new approaches can further our understanding of the occurrence of spontaneous synaptic events and provide quantitative tools to study synaptic transmission in AD and other disease models.

## Materials and Methods

### Animal

All animal use was approved by the University of California, Los Angeles Chancellor’s Committee on Animal Welfare.

*App^NL-F^* homozygous mice (AD; 18.3 ± 2.2 months; *n* = 8, three males and five females) were originally obtained from the research group led by Dr. Takaomi C. Saido at the Laboratory for Proteolytic Neuroscience, RIKEN Brain Science Institute, Saitama, Japan (Nilsson et al., 2014; Saito et al., 2014). Wild-type control mice (WT; 20.5 ± 3.2 months; *n* = 6, four males and two females) were either aged littermates from the *App^NL-F^* mice breeding or obtained from the National Institute on Aging. Mice were held in the vivarium on a 12/12 h light/dark cycle with free access to water and food.

### Electrophysiology

#### Slice preparation

Mice were anesthetized by isoflurane through inhalation, followed by rapid decapitation. Coronal acute brain slices were prepared on a Vibratome at 350-μm thickness in ice-cold cutting solution (135 mM NMDG, 10 mM D-glucose, 4 mM MgCl_2_, 0.5 mM CaCl_2_, 1 mM KCl, 1.2 mM KH_2_PO_4_, 20 mM HEPES, 3 mM kynurenic acid; ∼20 sucrose to adjust the osmolality; 305–310 mmol/kg; pH 7.35; bubbled with 100% O_2_). Then, slices were kept on a floating mesh platform in a recovery chamber filled with sucrose-based artificial CSF (sACSF; 55 mM sucrose, 85 mM NaCl, 25 mM D-glucose, 2.5 mM KCl, 1.25 mM NaH_2_PO_4_, 0.5 mM CaCl_2_, 4 mM MgCl_2_, 26 mM NaHCO_3_; 300–305 mmol/kg; bubbled with 95%O_2_/5% CO_2_). The recovery chamber was kept in 34°C water bath for 30 min and then moved to room temperature.

#### Whole-cell recording

For whole-cell recording, ACSF was used (126 mM NaCl, 10 mM D-glucose, 2 mM MgCl_2_, 2 mM CaCl_2_, 2.5 mM KCl, 1.25 mM NaH_2_PO_4_, 1.5 mM sodium pyruvate, 1 mM L-glutamine; 295–300 mmol/kg; bubbled with 95%O_2_/5% CO_2_). Acute brain slices were placed in a submerged chamber filled with ACSF at a flow rate of 6–7 ml/min and a temperature of 32–34°C. An intracellular solution (140 mM cesium methanesulfonate, 5 mM CsCl, 2 mM MgCl_2_, 2 mM Na-ATP, 2 mM Na-GTP, 200 μM EGTA, 10 mM HEPES; 275–280 mmol/kg; pH 7.35) was used for the recording of spontaneous synaptic events.

### Data acquisition and analysis

Spontaneous excitatory and inhibitory postsynaptic currents (sEPSCs and sIPSCs) were recorded at a holding voltage of -60 and 0 mV, respectively, using an Axopatch 200B amplifier (Molecular Devices, Inc.). All recordings were low-pass filtered at 1.5 kHz and digitized at a sampling frequency of 10 kHz, then recorded using a custom-written LabVIEW-based software (EVAN). Spontaneous events were detected with EVAN for the analysis of frequency and amplitude. The measurement of phasic discharge for the E/I ratio calculation was done using Igor Pro 6.3.7.2 (WaveMetrics, Inc.). The Igor code for the procedure will be provided on request. Other analyses on the synaptic events were done in Excel 2011 (Microsoft Corporation) with customized and built-in functions. Nested one-way ANOVA test was used to analyze E/I ratio differences, and it was done with an Excel spreadsheet from an online source (http://www.biostathandbook.com/nestedanova.html). All other statistical tests were done in Prism 6 (GraphPad Software, Inc.) by using non-parametric Mann–Whitney test or paired Wilcoxon test, and *p* < 0.05 was considered to be statistically significant. All the group statistics are presented as mean ± SEM (standard error of mean).

### Separating small and large events

To identify the large amplitude sEPSC and sIPSC events from all detected synaptic events, we used an approach that could objectively calculate a threshold for large events based on the amplitude distribution of all events from a cell. The cumulative distribution of all amplitudes was plotted and fitted with either one or two cumulative normal distributions using the NORM.DIST function in Excel.

The goodness of the two fits was compared with an *F* test. The *F* value was calculated as:

(1) (RSS1-RSS2)(p2-p1)RSS2(n-p2)

In Equation 1, *RSS_1_* and *RSS_2_* are the residual sum of squares when fitting with one and two distributions, respectively; *p_1_* and *p_2_* are the number of parameters used for fitting with one and two distributions, respectively; and *n* is the number of data points used for fitting. Then, a *p* value was calculated using the F.DIST function in Excel. For all the recordings in both groups, the *p* values approached 0, indicating that two distributions always fit the cumulative curves significantly better than one distribution. Next, a threshold for detecting large PSCs was chosen as the *x* value (amplitude) that corresponds to the *y* value (cumulative distribution) where the first distribution ends.

### Burstiness and memory calculation

The interevent intervals (IEIs) of synaptic events were measured as the time difference between two adjacent PSCs. Then, burstiness and memory of these events were calculated with equations in previous published papers (Goh and Barabási, 2008; Schleiss et al., 2016):

(2) $$B=\frac{\sigma -\mu }{\sigma +\mu }$$

(3) $$M=\rho_{IEI}(1)$$

In Equation 2, *B* is burstiness; and σ and *μ* are the standard deviation (SD) and mean of the IEIs, respectively, of each cell. In Equation 3, *M* is memory; and $\rho_{IEI}(1)$ is the Spearman rank auto-correlation of the IEI array at a lag of 1.

### Calculation of E/I ratio

#### Measurement of phasic charge

We have previously described an approach to objectively measure the tonic and phasic components of inhibitory or excitatory events (Glykys and Mody, 2007). This method does not rely on subjective thresholds to detect spontaneous events. For the analysis of E/I ratio based on this approach, the raw recording traces of certain durations were analyzed. Using a custom-written Igor procedure, an all-point histogram (bin width = 1 pA) was plotted for segments of 15 s from either sEPSC or sIPSC recordings and smoothed by Savitzky–Golay algorithm (2nd order 17 points smoothing) to obtain the peak value. This histogram was always skewed to one side by the presence of spontaneous synaptic events. The non-skewed side of the histogram was fitted with a Gaussian distribution from the end of the non-skewed side to 95% of the peak value over the peak to the skewed side. The mean of this fitted Gaussian distribution was considered to be the mean holding current. Then, this Gaussian curve was mirror-imaged to the skewed side, and the area between the Gaussian distribution and the all-point histogram divided by the total number of points in this area was considered to be the mean phasic charge (in pC). The E/I was determined as the ratio between the excitatory and the inhibitory phasic charges over 15-s recordings. For each cell, we have randomly selected 10 segments from sEPSC and sIPSC recordings (see below for details) and obtained 100 E/I values.

#### Driving forces for sEPSCs and sIPSCs

To get a more accurate measurement of the E/I ratios, we have also taken the driving force for sEPSCs and sIPSCs into account. The holding voltages for recording sEPSCs and sIPSCs were -60 and 0 mV, respectively. The reversal potentials of the currents activated by AMPA and NMDA are close to 0 mV, and that calculated for the GABA receptors is approximately -70 mV. Therefore, at V_h_ = -60 mV, the driving forces for sEPSCs and sIPSCs were ∼60 and 10 mV, respectively; whereas at V_h_ = 0 mV, the driving forces for sEPSCs and sIPSCs were ∼0 and 70 mV, respectively. At V_h_ = -60 mV where sEPSCs were recorded, it is possible that the recordings were contaminated with small outward sIPSCs. Because this contamination should be absent in picrotoxin (PTX), for some recordings at V_h_ = -60 mV, we plotted the all-point histograms of one randomly chosen 15 s segment before and one after washing in 50 μM PTX. We then compared the SD (σ) of the Gaussian fits of the outward currents (non-skewed sides, as described above) of the two histograms. A significant amount of small contaminating sIPSCs in the sEPSC recording would significantly decrease the σ after PTX. In total, we have got three WT and seven AD cells with PTX wash in at -60 mV, and the variance before and after PTX was not significantly different in these 10 cells (WT: σ = 3.92 vs 3.79; *p* = 0.75; AD: σ = 2.54 vs 3.06; *p* = 0.22; before vs after PTX; Wilcoxon paired test). Therefore, there was no detectable contamination of small outward sIPSCs in our sEPSC recordings at V_h_ = -60 mV.

#### Determination of sample size

With the approach above, we can get the phasic charge by randomly choosing *m* segments from sEPSC recording and *n* segments from sIPSC recording for each cell, and then get *m × n* E/I ratios. To determine the minimum *m* and *n* value needed to get the best estimation of the real population E/I value, we used the equation described in a previously published paper (Eckblad, 1991) for estimating the fractional deviation of the sample mean from the real population mean:

(4) $$Fractional\;deviation=\frac{t\times \sigma }{\mu \times \sqrt{N}}$$

In Equation 4, *t* is the value from a Student’s *t* distribution with a degree of freedom equal to one less than the sample size and with a 95% confidence interval; σ is the sample SD; *μ* is the sample mean; and *N* is the sample size.

Firstly, we have determined a way to randomly select segments of recording from a trace. Random integer numbers between the start and end time (in s) of the recording were generated successively using the RANDBETWEEN function in Excel, and the random numbers will be used as the start time of different segments. If two segments had an overlap, then the start time of the latter-generated segment was regenerated. If one segment ran into the seal test period, the start time was also regenerated. In this way, we have randomly selected 10 segments of 15 s from sEPSC and sIPSC recording for each cell, yielding 100 E/I ratios (*N* = 100). We then randomly selected different number of values (*N* = 10, 20, 30, …, 90) from these 100 values for calculating the σ and *μ* of different sample sizes. With Equation 4, we calculated the fractional deviation of different sample sizes (*N* = 10, 20, …, 100) for each cell. We also calculated the fractional deviation using the σ and *μ* of *N* = 100 for all sample sizes and estimated the fractional deviation of more sample sizes. With this approach applied to every cell, we found that when *N* = 100, the fractional deviation of the sample mean from the real population mean reached a sufficiently low level. Therefore, considering the accuracy of estimating the real E/I ratio and the convenience of choosing samples, we decided to choose 10 segments from sEPSC recording and 10 from sIPSC recording, thus obtaining all possible 100 E/I values for each cell.

#### Statistical test for phasic charges and E/I ratios

Since we have recordings from different animals, and for each animal we had recording(s) from one or multiple cells, we want to take all variables into account when we compare the Es and Is as well as the E/I ratios between groups. Therefore, we decided to use a nested one-way ANOVA to determine the difference between the WT and AD group. The test was done using an Excel spreadsheet from an online source (http://www.biostathandbook.com/nestedanova.html). Briefly, there were groups (WT and AD), subgroups (for a two-level nested ANOVA), and sub subgroups (for a three-level nested ANOVA) in this test. The phasic E and I charges of WT and AD cells were compared by a two-level nested one-way ANOVA, in which each animal was treated as a subgroup, and the average of the 10 Es and 10 Is of each cell from this animal was listed as values within the subgroup. Then, the *p* values of the difference between groups (WT and AD) as well as among subgroups within each group (six and eight mice in the WT and AD group, respectively) were calculated. The E/I ratios of WT and AD cells were compared by a three-level nested one-way ANOVA, because we did not want to use the average of 100 E/I ratios as a single value for each cell and lose the accuracy (see the determination of sample size). Therefore, besides groups (WT and AD) and subgroups (different animals), there were also sub subgroups, which are different cells from each animal, and then the 100 E/I ratios were listed as values within the sub subgroups. Similarly, the *p* values of the difference between groups (WT and AD), among subgroups within each group (six mice in the WT group; eight mice in the AD group), as well as among sub subgroups within each subgroup (9 and 12 cells in the WT and AD group, respectively) were calculated.

## Results

### Reduced sIPSC amplitudes in App^NL-F^ mice

**Figure 1.**
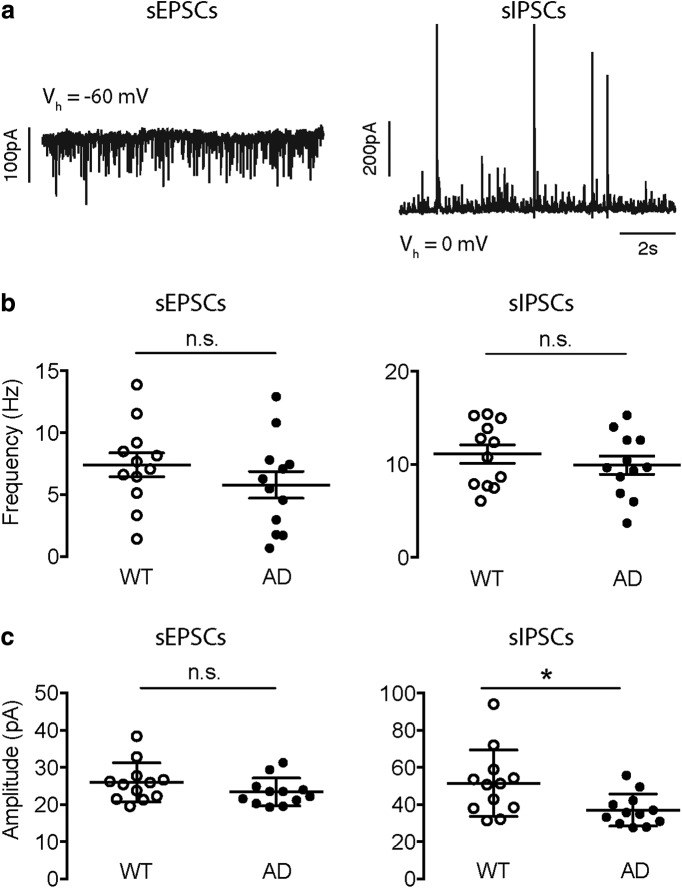
The average amplitude of sIPSCs was reduced in AD pyramidal cells. ***A***, Example raw recording traces of sEPSCs (left) and sIPSCs (right), with a holding voltage (V_h_) of -60 and 0 mV, respectively. ***B***, The average frequency of either sEPSCs (left) or sIPSCs (right) remained unchanged in the AD group. ***C***, The average amplitude of sEPSCs remained constant (left), while that of sIPSCs was significantly decreased (right) in the AD group. *p < 0.05, n.s.: not significant.

Previous studies have shown altered glutamatergic and GABAergic transmission in parietal cortex in various mouse models of AD (Busche et al., 2008; Roberson et al., 2011; Verret et al., 2012; Hazra et al., 2013; Perez et al., 2016). We wanted to investigate whether there are alterations in excitatory and inhibitory synaptic transmission in the *App^NL-F^* mouse model of AD. We have recorded sEPSCs and sIPSCs (see Materials and Methods; Fig. 1*A*) in parietal cortex layer 2/3 pyramidal cells in both WT (*App^wt/wt^*; WT) and *App^NL-F^* homozygous (*APP^NL-F/NL-F^*; AD) mice. We chose to study synaptic transmission in the parietal cortex because in this region, altered synaptic transmission and aberrant network activities were observed in hAPP mice (Roberson et al., 2011; Verret et al., 2012), and atrophies and hypoperfusion were observed in AD patients (Jacobs et al., 2011, 2012). The average ages were comparable between the two groups (20.5 ± 3.2 vs 18.3 ± 2.2 months; *n* = 6 vs 8 mice; WT vs AD; *p* = 0.5088; Mann–Whitney test). For each cell we analyzed the same number of consecutively occurring sEPSCs or sIPSCs. The events were randomly chosen from all detected events throughout the whole recording period (12 cells for each group; 161 sEPSCs and 626 sIPSCs for each cell). The average frequencies of both sEPSCs and sIPSCs remained unchanged in the AD mice (sEPSC frequency = 7.42 ± 0.97 vs 5.79 ± 1.08 Hz, *p* = 0.2415; sIPSC frequency = 11.10 ± 0.99 vs 9.91 ± 0.98 Hz, *p* = 0.2415; WT vs AD; Mann–Whitney test; Fig. 1*B*). The average amplitude of sIPSCs was significantly reduced while that of sEPSCs remained constant in the AD animals (sEPSC amplitude = 25.97 ± 1.52 vs 23.39 ± 1.07 pA, *p* = 0.1432; sIPSC amplitude = 51.55 ± 5.16 vs 37.06 ± 2.50 pA, *p* = 0.0145; WT vs AD; Mann–Whitney test; Fig. 1*C*). Since we were using WT mice from two different sources as control, either from littermates in the *App^NL-F^* colony (three cells; two mice) or from NIA (nine cells; four mice), we have examined whether there was a significant difference within the two subtypes of our controls. Statistical analysis revealed no significant difference either in the frequency and amplitude of sEPSCs (*p* = 0.6000 and *p* = 0.4818; sEPSC frequency and amplitude, respectively; littermates vs NIA; Mann–Whitney test) or in those of sIPSCs (*p* = 0.6000 and *p* = 0.4818; sIPSC frequency and amplitude, respectively; littermates vs NIA; Mann–Whitney test) between the *App^NL-F^* littermates and the NIA mice.

### The reduction of sIPSC amplitudes in App^NL-F^ mice was caused by a decrease in large amplitude events

The presence of large amplitude events during sIPSC recording led us to further investigate whether small and large events contributed differently to the overall changes in the amplitudes of sPSCs. We were specifically interested in analyzing the large sIPSCs because they potentially originate from perisomatic-targeting PV INs which generate strong inhibition on postsynaptic neurons (Freund and Katona, 2007). Since previous research has shown a deficiency in the intrinsic excitability of GABAergic PV INs in hAPPJ20 mouse model of AD (Verret et al., 2012), we wanted to examine whether the reduction in the sIPSC amplitudes we observed could be related to PV IN dysfunction in the novel *App^NL-F^* AD model.

**Figure 2.**
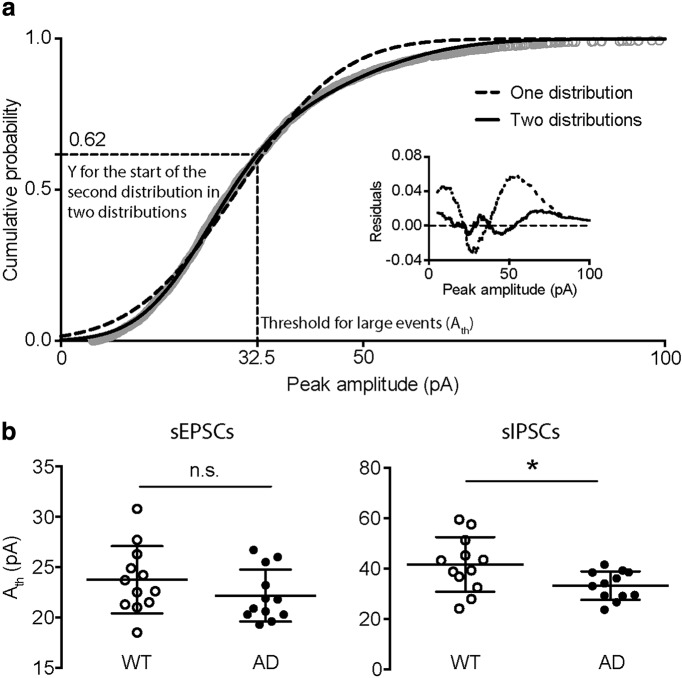
The sEPSCs and sIPSCs from each cell can be divided into two groups according to the bimodal distribution of their amplitudes. ***A***, Example of choosing an objective amplitude threshold (A_th_) to divide the sIPSCs into small and large groups. The cumulative distribution of all sIPSC amplitudes was fitted with either one (dashed line) or two (solid line) cumulative normal distributions. When fitting with two distributions, the amplitude threshold (A_th_) is the corresponding X value on the fitted curve at the start of the second distribution. Inset, the difference between the predicted and the actual amplitude values (residuals) when fitting with one (dashed line) or two (solid line) cumulative normal distributions. ***B***, The A_th_ of sEPSCs and sIPSCs in WT and AD cells. The A_th_ of sEPSCs remained constant (left), while that of sIPSCs was reduced (right) in AD cells. *p < 0.05, n.s.: not significant.

To study the small and large sPSCs separately, we first tested whether the events could be objectively divided into two groups (see Materials and Methods). We found that the cumulative amplitude distributions of all sPSCs for each individual cell could be significantly better fitted by two cumulative normal distributions rather than one, as indicated by an *F* test (see Materials and Methods; data not shown), thus sPSCs in all cells could be separated into two groups according to their amplitudes (Fig. 2*A*). Based on the fitted curve, we chose to use the value on the *x*-axis (amplitudes) corresponding to the end of the first distribution on the *y*-axis (cumulative probability) as the threshold for large events (A_th_; Fig. 2*A*). Then, sPSCs larger than A_th_ were classified as large events, while those smaller than A_th_ were considered as small events. The averaged A_th_ for sEPSCs was comparable between WT and AD, while for sIPSCs the averaged A_th_ was significantly decreased in AD mice (sEPSC A_th_ = 23.75 ± 0.96 vs 22.18 ± 0.75 pA, *p* = 0.1735; sIPSC A_th_ = 41.64 ± 3.14 vs 33.24 ± 1.64 pA, *p* = 0.0332; WT vs AD; Mann–Whitney test; Fig. 2*B*), which is consistent with the reduced amplitude of sIPSCs (Fig. 1*C*).

**Figure 3.**
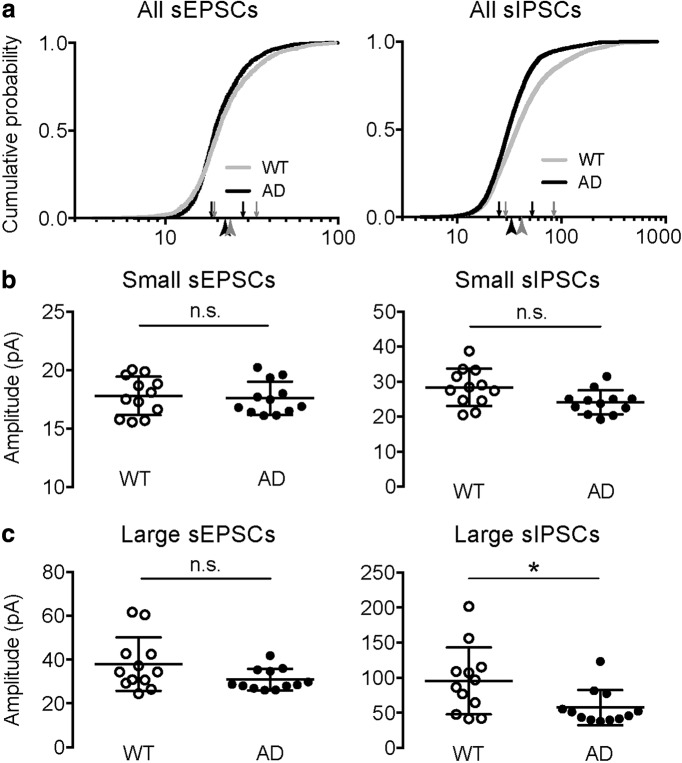
The average amplitude of large sIPSCs was significantly reduced in AD pyramidal cells. ***A***, The cumulative distributions of sEPSC (left) and sIPSC (right) amplitudes of all cells recorded in WT (gray curve) and AD (black curve) mice. The arrows are showing the mean of the first and second distribution when fitting with two cumulative normal distributions for WT (gray arrows) and AD (black arrows) cells. The arrowheads are showing the A_th_ values for WT (gray arrowheads) and AD (black arrowheads) cells. ***B***, The average amplitudes of small sEPSCs (left) and small sIPSCs (right) were both not significantly altered in the AD group. ***C***, The average amplitude of large sEPSCs was unchanged (left), while that of large sIPSCs was significantly reduced (right) in the AD group. *p < 0.05, n.s.: not significant.

From the cumulative distribution curves of all sPSCs detected from all cells in WT and AD mice, qualitatively the amplitude distribution of sEPSCs were close between WT and AD group, while the distribution curve of sIPSCs of AD mice were significantly left-shifted compared to that of WT mice (Fig. 3*A*), which is consistent with a decreased average sIPSC amplitude in AD animals (Fig. 1*C*). Moreover, the two curves of sIPSC amplitudes became further separated when the events were larger than A_th_ (Fig. 3*A*, arrowheads), revealing a more significant reduction in large sIPSCs than in small sIPSCs. When small and large events were analyzed separately, the amplitudes of both large and small sEPSCs remained constant (small sEPSC amplitude = 17.80 ± 0.48 vs 17.60 ± 0.41 pA, *p* = 0.7987; large sEPSC amplitude = 37.90 ± 3.52 vs 30.81 ± 1.43 pA, *p* = 0.1135; WT vs AD; Mann–Whitney test; Fig. 3*B*,*C*), which is consistent with the unchanged overall sEPSC amplitude (Fig. 1*C*). For sIPSCs, the amplitude of large sIPSCs was decreased in AD mice while that of the small sIPSCs remained unchanged (small sIPSC amplitude = 28.35 ± 1.54 vs 24.07 ± 1.00 pA, *p* = 0.0519; large sIPSC amplitude = 95.36 ± 13.81 vs 57.31 ± 7.27 pA, *p* = 0.0284; WT vs AD; Mann–Whitney test; Fig. 3*B*,*C*), consistent with a decrease in the overall sIPSC amplitude (Fig. 1*C*) and the trend in the distribution curves (Fig. 3*A*).

Therefore, we have revealed that there are two populations in all sPSCs, and that they can be separated by an objectively calculated amplitude threshold. Further, we have demonstrated that the decrease in the overall sIPSC average amplitude was primarily contributed by the large events rather than the small ones, which is consistent with our PV IN dysfunction hypothesis in the *App^NL-F^* mice.

### Rates of rise of sIPSCs were decreased in App^NL-F^ mice

**Figure 4.**
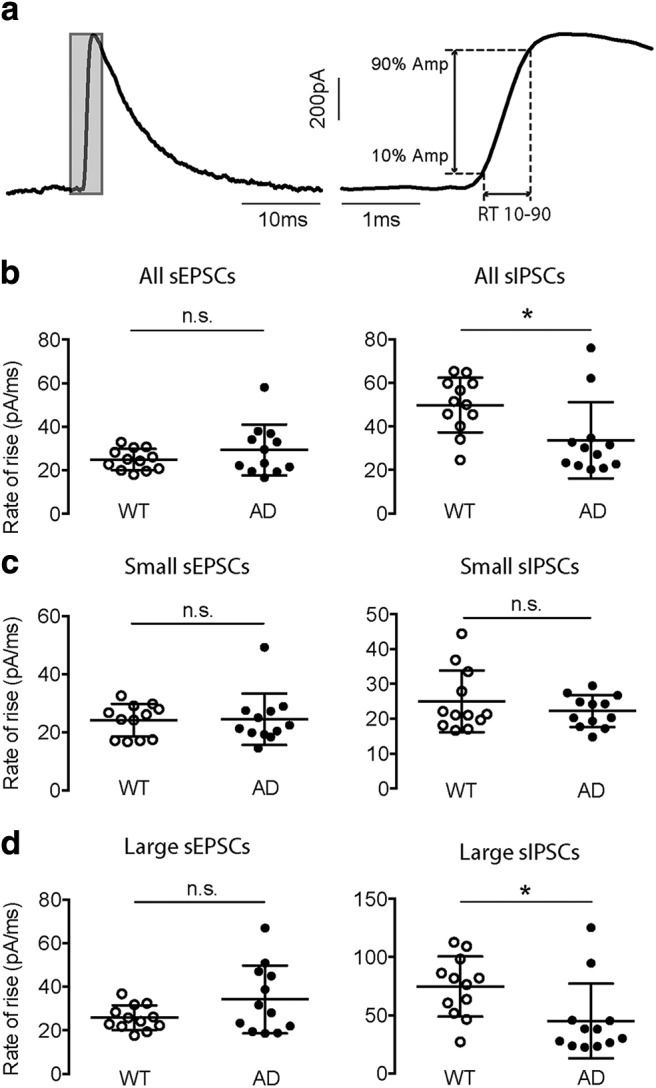
The analysis of RR revealed a significant decrease only in the large sIPSCs of AD pyramidal cells. ***A***, The RR was calculated as the ratio between the increase in current and the rise time (RT) from 10% (10% Amp) to 90% (90% Amp) of the peak. ***B***, The average RR of all sEPSCs was unchanged (left), while that of all sIPSCs was reduced (right) in the AD group. ***C***, The average RR of either small sEPSCs (left) or small sIPSCs (right) was not altered in the AD group. ***D***, The average RR of large sEPSCs remained constant (left), while that of large sIPSCs was significantly reduced (right) in the AD group. *p < 0.05, n.s.: not significant.

The decreased amplitudes of large sIPSCs in *App^NL-F^* mice is consistent with a PV-IN dysfunction. However, the origin of the large sIPSCs needs to be further analyzed, since they may be a summation of multiple events (Williams et al., 1998), or they could be generated by other types of perisomatic-targeting interneurons (Freund and Katona, 2007). Since PV-INs target the α1 subunit-containing GABA_A_ receptors, they mainly generate fast-rising PSCs (Thomson et al., 2000; Nyíri et al., 2001; Klausberger et al., 2002). To further elucidate the origination of the large sIPSCs, we also examined the RR of the synaptic currents. We defined the RR of an event as the ratio between 80% of the peak amplitude and RT 10-90, which is the time it takes for the current to rise from 10% to 90% of the peak amplitude (Fig. 4*A*). Thus, the RR of an event is the slope of the main rising phase and indicates how fast an event rises to its peak. We found that the RRs of sEPSCs was unchanged in the AD group, while those of sIPSCs was significantly decreased in AD animals (sEPSC RR = 24.92 ± 1.41 vs 29.33 ± 3.37 pA/ms, *p* = 0.5137; sIPSC RR = 49.82 ± 3.62 vs 33.63 ± 5.06 pA/ms, *p* = 0.0100; WT vs AD; Mann–Whitney test; Fig. 4*B*).

When we separated the events based on their amplitudes into small and large ones, the results showed that the RRs of small sEPSCs were comparable to those of the large ones for both WT and AD mice (*p* = 0.6707, WT; *p* = 1432, AD; Mann–Whitney test). Whereas for sIPSCs, the RRs of large events were significantly higher than those of the small ones in both groups (*p* < 0.0001, WT; *p* = 0.0029, AD; Mann–Whitney test). These results are consistent with the idea that large amplitude sIPSCs comprise events originating from soma-innervating PV INs that synapse onto α1 subunit-containing GABA_A_ receptors. Furthermore, the RRs of both small and large sEPSCs were comparable between WT and AD group (small sEPSC RR = 24.07 ± 1.63 vs 24.47 ± 2.56 pA/ms, *p* = 0.7987; large sEPSC RR = 25.76 ± 1.64 vs 34.18 ± 4.49 pA/ms, *p* = 0.3474; WT vs AD; Mann–Whitney test; Fig. 4*C*,*D*). For sIPSCs, while the RRs of the small events were similar, those of the large events were significantly decreased in AD mice (small sIPSC RR = 24.97 ± 2.55 vs 22.21 ± 1.33 pA/ms, *p* = 0.6707; large sIPSC RR = 74.67 ± 7.47 vs 45.06 ± 9.27 pA/ms, *p* = 0.0083; Mann–Whitney test; Fig. 4*C*,*D*), indicating again that the changes in large amplitude and fast-rising sIPSCs led to the overall reduction of RRs in AD mice.

In conclusion, our results from analyzing the RR further indicated that the large sIPSCs could be generated by soma-targeting PV INs according to their fast rising phase. Moreover, the reduction in the overall rates of rise in AD cells is contributed by large events but not the small ones, which is again consistent with a PV IN dysfunction.

### Burstiness and memory of sPSCs

Besides analyzing the frequency and amplitude of sEPSCs and sIPSCs, we also wanted to investigate whether there are specific firing patterns within those events, such as bursts. to examine the burstiness of sPSCs on pyramidal cells in WT and AD mice, we first determined the IEIs of all sPSCs as well as those of the large sPSCs. Then, we used previously described methods to quantitatively determine the burstiness (B) and memory (M) in the firing of a single cell (Goh and Barabási, 2008; Schleiss et al., 2016; see Materials and Methods). By default, the values of both burstiness and memory will be within the range (-1, 1). For burstiness, a value closer to 1 (i.e., when SD is very large) means the firing pattern is more “bursty,” while a value closer to -1 (i.e., when SD is very low) indicates a more regular firing pattern. In between, a Poisson process will result in a burstiness value of 0. For memory, a positive value means that short IEIs tend to be followed by short IEIs and long IEIs by long IEIs, whereas a negative value means that short IEIs tend to be followed by long IEIs or vice versa. A value near 0 indicates no memory in the system.

**Figure 5.**
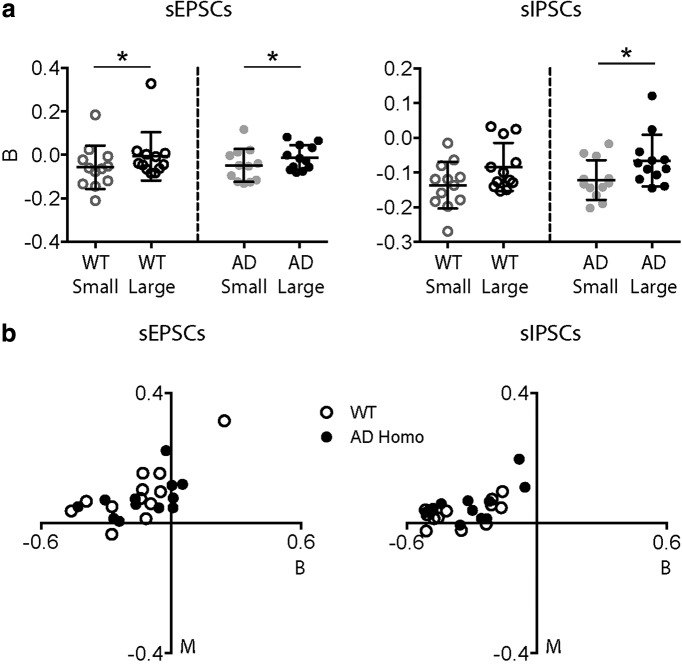
The analysis of burstiness (*B*) and memory (*M*) showed different firing patterns of small and large events. ***A***, Left, The burstiness of large sEPSCs was significantly closer to 0 (indicating a Poisson process) than that of small sEPSCs for both WT and AD pyramidal cells. Right, the burstiness of large sIPSCs was significantly closer to 0 than that of small sIPSCs for AD cells, but not for WT cells. ***B***, The distribution of *B* and *M* values calculated with all sEPSCs (left) and sIPSCs (right) from each cell in a *B-M* plane showed negative B values, indicating regular firing pattern, and close-to-zero M values, indicating little memory in both WT and AD pyramidal cells. *p < 0.05.

The results from burstiness analysis showed that for sEPSCs and sIPSCs in both WT and AD cells, the average values were all negative (B_EPSC_WT_ = -0.19 ± 0.04; B_IPSC_WT_ = -0.35 ± 0.04; B_EPSC_AD_ = -0.13 ± 0.04; B_IPSC_AD_ = -0.31 ± 0.04), indicating that the occurrence of sPSCs was more regular than bursty. Also, there was no significant difference in burstiness either for sEPSCs or for sIPSCs between WT and AD cells (data not shown). However, when we compared the burstiness of small events versus that of large events, we found that in general, small sPSCs had more negative B values than large sPSCs for both WT (B_Small EPSC_ = -0.06 ± 0.03 vs B_Large EPSC_ = -0.01 ± 0.03; B_Small IPSC_ = -0.14 ± 0.02 vs B_Large IPSC_ = -0.08 ± 0.02; Fig. 5*A*) and AD (B_Small EPSC_ = -0.05 ± 0.02 vs B_Large EPSC_ = -0.01 ± 0.02; B_Small IPSC_ = -0.12 ± 0.02 vs B_Large IPSC_ = -0.07 ± 0.02; Fig. 5*A*) mice, and that difference was significant for sEPSCs of both WT and AD cells (*p* = 0.0425 for WT; *p* = 0.0425 for AD; Wilcoxon paired test; Fig. 5*A*) and for sIPSCs of AD cells only (*p* = 0.0522 for WT; *p* = 0.0122 for AD; Wilcoxon paired test; Fig. 5*A*). These results indicate that for both sEPSCs and sIPSCs, the small events occurred regularly throughout the recordings, while the large events were generated in a more random pattern. For memory, the averaged memory for all sEPSCs and all sIPSCs was close to 0 (M_EPSC_WT_ = 0.07 ± 0.02; M_IPSC_WT_ = 0.03 ± 0.01; M_EPSC_AD_ = 0.08 ± 0.02; M_IPSC_AD_ = 0.06 ± 0.02), indicating that there is little memory in the system. Also, there was no significant difference in memory either between small and large events or between WT and AD groups (data not shown).

According to the original study (Goh and Barabási, 2008), the “burstiness” of a system can have two qualitatively different origins, which are presented by parameters B and M described above. Therefore, it is helpful to place B and M values in a *(B, M)* space which can give an intuitive presentation of certain properties of a system, which, in our case, is the pattern of incidence of sPSCs (Fig. 5*B*). From the *(B, M)* plots of cell averaged memory and burstiness of sEPSCs and sIPSCs in WT and AD group, it is evident that both currents lack memory and are not bursty. On the contrary, the sPSCs appeared to emerge in a rather regular pattern.

### A novel approach for determining the synaptic E/I ratio

A proper balance between excitation (E) and inhibition (I) is difficult to define consistently under all circumstances (Isaacson and Scanziani, 2011), but it is considered to be crucial for a normal circuit function. Studies have shown that a disrupted E/I balance can cause network dysfunction and various diseases (Fernandez et al., 2007; Kehrer et al., 2008; Gogolla et al., 2009), including AD (Schmitt, 2005; Rissman and Mobley, 2011; Busche and Konnerth, 2016). Therefore, after examining the excitatory and inhibitory currents separately in pyramidal cells, we also wanted to check whether the E/I balance is altered in *App^NL-F^* mice. Previous methods have been using multiple indices to calculate the E/I ratio, such as the peaks, charges (Bartley and Dobrunz, 2015) and conductance (Wehr and Zador, 2003; Cruikshank et al., 2007) of PSCs. These existing methods usually use only one averaged property of the events, and they usually acquire the E and I value over a short period of recording. Here, we introduce a new approach, which can incorporate all properties of sPSCs into a single index and is calculated multiple times over a long duration of recording to determine the E/I ratio.

Our previous work has described a method to objectively separate the tonic and phasic components from raw electrophysiological recordings (Glykys and Mody, 2007; see Materials and Methods). There are several advantages of using this method to determine the phasic E and I exerted onto a cell: firstly, no subjective thresholds are needed to detect spontaneous events, and tonic and phasic activities are separated objectively according to the all-point histograms of raw recording traces; secondly, the values of E and I depend on all properties of the phasic currents, such as frequency, amplitude, charge, etc.; and thirdly, it allows the selection of multiple segments from the entire recording and acquire a vast number of E/I values.

**Figure 6.**
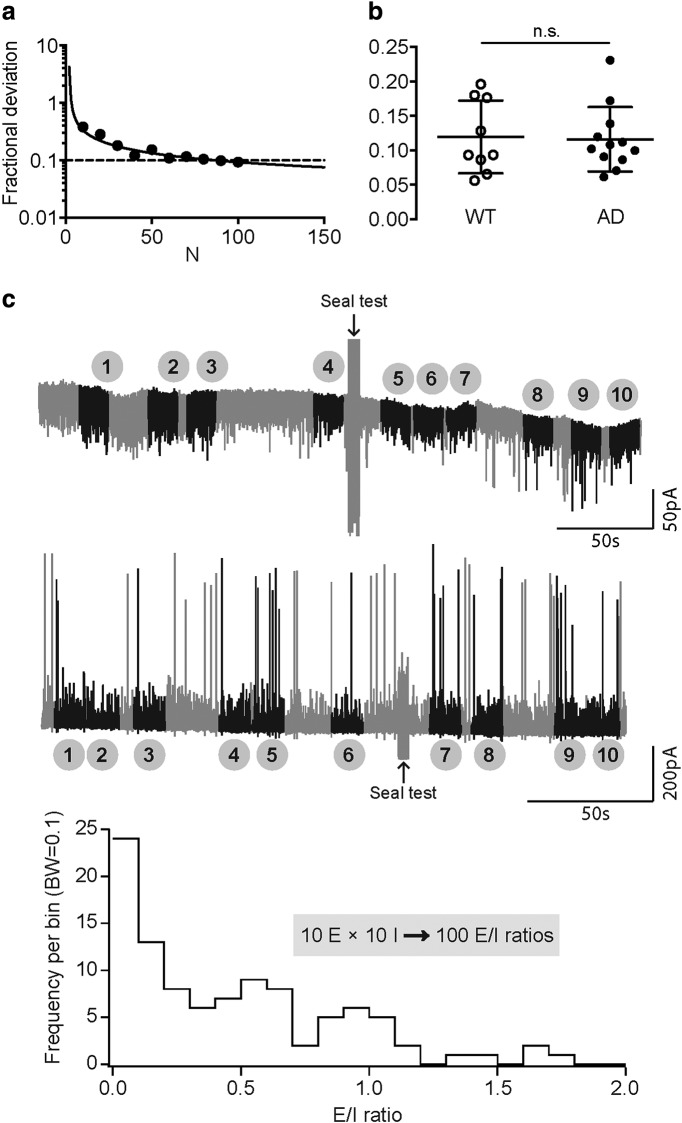
A new approach to determine the E/I ratio can predict the real E/I value of each cell with high accuracy. ***A***, The fractional deviation of the sample mean from the real population mean decreases with the increase of sample size (N). The curve shows deviations calculated with the mean (*μ*) and SD (*σ*) of 100 samples (*N* = 100), and the 10 dots are deviation values calculated with the *μ* and *σ* of *N* = 10, 20, …, 100 samples, from left to right. ***B***, When acquiring 100 E/I ratios, for both WT and AD cells, the fractional deviation reaches a low value (∼12%), and it is not significantly different in the two groups. ***C***, Randomly selecting 10 segments of 15 s from the raw recording traces of sEPSCs (top) and sIPSCs (middle) can yield 100 E/I ratios when crossing over the 10 phasic E and 10 phasic I charges (bottom). n.s.: not significant.

We decided to randomly choose *m* segments from sEPSC recordings (E) and *n* segments from sIPSC recordings (I) for each cell, and each segment with a duration of 15 s. Then, we will have *m* E and *n* I values, resulting in *m × n* E/I ratios. To determine the proper values for *m* and *n*, we did a sample size analysis using a previously described method (Eckblad, 1991; see Materials and Methods). The fractional deviation of the sample mean from the real population mean is negatively correlated with the sample size (Fig. 6*A*). For the cell shown in Figure 6*A*, when the number of E/I ratios reached 100, the fractional deviation decreased to 0.09, which means that the sample mean is ±9% within the real population mean. Next, we did the same analysis for each cell in the WT and AD groups. The results showed that when calculating 100 E/I ratios, the fractional deviation in predicting the real population mean would be sufficiently low (F_WT_ = 0.12 ± 0.02 vs F_AD_ = 0.12 ± 0.01, p > 0.9999; *n* = 9 vs *n* = 12; WT vs AD; Mann–Whitney test; Fig. 6*B*). Therefore, for each cell, we decided to randomly select 10 segments of 15 s from both the E and I raw recording traces, thus obtaining 10 E and 10 I values and 100 E/I ratios for one cell (Fig. 6*C*).

**Figure 7.**
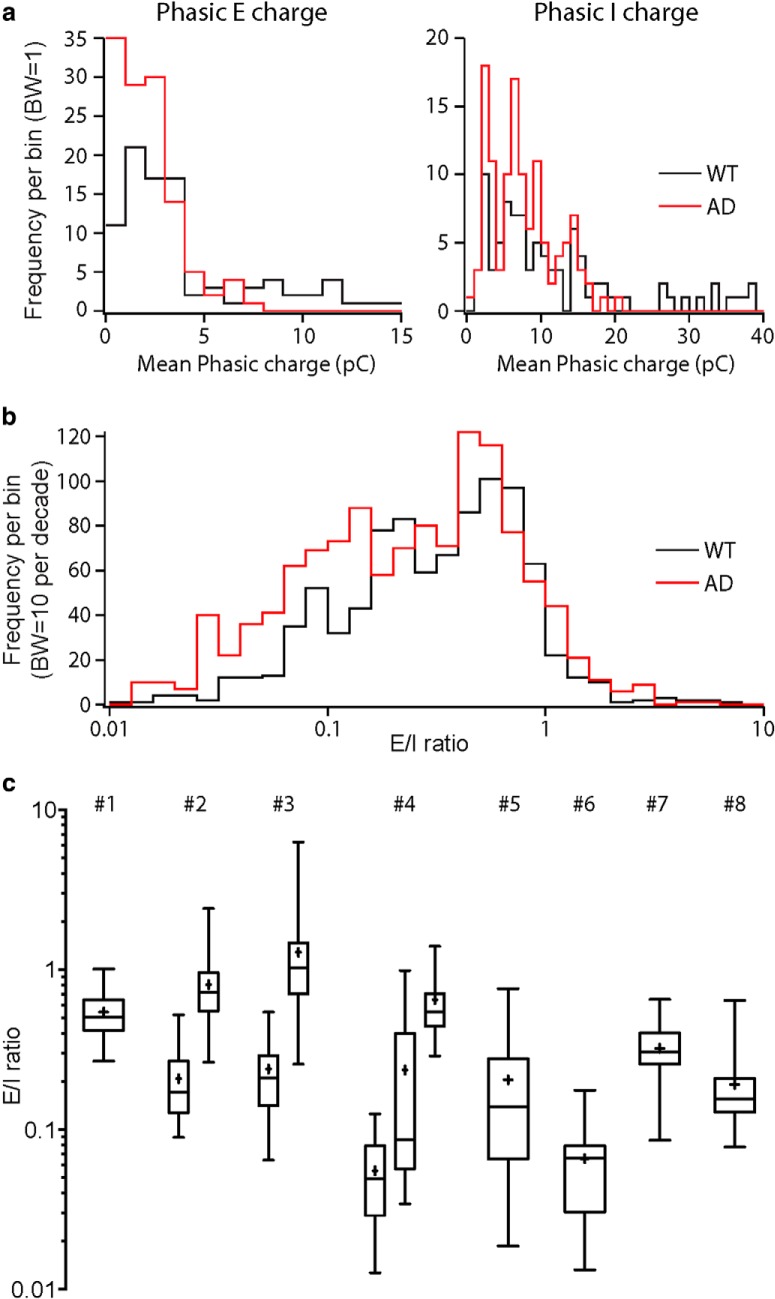
The E/I ratios calculated with the new approach showed a tendency of reduction in the AD group. ***A***, Histogram of phasic E (left) and phasic I (right) charges showed a reduction in both E and I in AD pyramidal cells (10 E and 10 I values for each cell). ***B***, Histogram of E/I ratio showed a shift to the left in the AD group, however the E/I was not significantly altered according to a nested two-way ANOVA test. ***C***, The E/I values for the AD group showed large variations among the 100 values in each cell (shown as each bar), among different cells in each animal (shown as each #), and among different animals. Box: 25th to 75th percentiles, line in the box: median, whiskers: min to max, +: mean.

We first examined the phasic E and I values in WT and AD cells. As described above, each group there will be *N × 10* values of E and I, in which *N* is the number of cells in each group (N_WT_ = 9; N_AD_ = 12). Considering that these values were collected from different animals (six and eight mice in the WT and group, respectively) and different cells (9 and 12 cells in the WT and AD group, respectively), besides comparing the average of all values in each group, we also included the variance among different animals and cells into the analysis. Therefore, we decided to use a two-level nested one-way ANOVA test (see Materials and Methods). The results showed that there was no significant difference either between groups (p_Es_ = 0.1276; p_Is_ = 0.2602; WT vs AD), or among subgroups within each group (p_Es_ = 0.2080; p_Is_ = 0.0973; among animals). The results from analyzing detected PSCs indicated a significant difference in the average sIPSC amplitude but not the average sEPSC amplitude between groups (Fig. 1*C*). The absence of significance in the phasic I charge analysis may be caused by the large variance among subgroups, i.e., among different animals (31.98% and 52.83% of total variance, Es and Is, respectively), as well as within subgroups, i.e., among different cells (53.15% and 44.70% of total variance, Es and Is, respectively). In comparison, the variance between the WT and AD group was smaller (14.87% and 2.47% of total variance, Es and Is, respectively). Moreover, in the phasic charge analysis, when there were no subjective detection thresholds chosen, many more very small events (below detection threshold) contributed to the phasic component of a recording. As we have shown above, the large events but not the small ones contributed to the overall difference in amplitudes (Fig. 3*B*,*C*) as well as rates of rise (Fig. 4*C*,*D*) between groups. Therefore, the considerably more small events below detection threshold contributing to the phasic charge resulted in the overall unchanged phasic E and I; however, in the histograms of all the values, we could see a left-shift in both Es and Is in the AD group (Fig. 7*A*).

For the calculation of the E/I ratio, firstly we’ve taken into account the driving forces for sEPSCs and sIPSCs. In our recording scenario, the driving forces for sEPSCs and sIPSCs were ∼60 and 70 mV, respectively (see Materials and Methods). Therefore, to counterbalance the difference in driving forces, the E/I ratio was determined as (7×E)/(6×I). To compare the E/I ratio between WT and AD animals, we chose to use a three-level nested one-way ANOVA test (see Materials and Methods), because now we want to use all the 100 E/I ratios for a higher accuracy, there was an extra level, sub subgroups, which were different cells from each animal. Then, the 100 values from each cell were listed under each sub subgroup. As a result of the unchanged phasic E and I values, the E/I ratio remained constant in the AD group as well (E/I = 0.54 ± 0.02 vs 0.47 ± 0.02, *p* = 0.3882), although there was a left-shift in the histogram of all E/I ratios in the AD group (Fig. 7*B*). Another reason that might result in the unchanged E/I ratio might be the large variance among different animals and cells. As an example, in the AD group, the E/I ratios varied a lot from animal to animal as well as among different cells in the same animal (Fig. 7*C*).

## Discussion

We investigated synaptic alterations by analyzing sPSCs in parietal cortex layer 2/3 pyramidal cells in the *App^NL-F^* mouse model of AD. Through objective amplitude distribution analysis, we found that while the properties of small sIPSCs and all sEPSCs remained constant, the amplitudes of large and fast-rising sIPSCs were reduced in AD mice, which is consistent with deficits in PV INs. To further characterize the properties of the spontaneous synaptic currents as part of a chain of successive events, we introduced the calculation of burstiness and memory into the analysis. We also used a novel method to determine the E/I ratio with a high accuracy. Owing to the large variance among different animals and cells, we found no significant alteration in the overall E/I ratio in AD mice. It remains to be determined whether homeostatic mechanisms or experimental conditions are responsible for the unchanged E/I ratio in *App^NL-F^* mice. Together, our results have shown the advantage of using various quantitative approaches in the field of synaptic physiology, and provided ground for future research in elucidating the synaptic and network properties of the *App^NL-F^* mouse model as well as in seeking potential therapeutic targets to treat symptoms associated with, and contributing to, the development of AD.

In our endeavor to thoroughly study synaptic changes in the cortices of *App^NL-F^* mice, we developed reliable and objective methods to analyze spontaneous synaptic events. One key element of our approach was to avoid using subjective and randomly chosen thresholds. For the separation of small and large synaptic events, we used the cumulative distribution of all event amplitudes to determine the threshold of separation between small and large amplitude currents. For obtaining the E/I ratio, we used the all-point histogram and Gaussian fits to calculate the phasic charges, where no subjective thresholds were needed for the detection of synaptic currents. While it is considered to be crucial for a normal circuit function, the E/I ratio remains difficult to define consistently under all circumstances (Isaacson and Scanziani, 2011). Existing methods have used one or multiple parameters of mainly stimulus-evoked PSCs, such as peak amplitudes and charges, over short periods of recording to determine the E/I ratio (Wehr and Zador, 2003; Cruikshank et al., 2007; Bartley and Dobrunz, 2015). Considering potential drawbacks of these methods, we developed an approach which could (1) incorporate all properties of the PSCs such as frequency, amplitude, charge, etc. and (2) increase the accuracy of E/I calculation by including multiple segments over relatively long periods of recording (4–6 min) since the E/I balance is dynamically changing in the brain.

In the *App^NL-F^* mice, using our new approach, the E/I ratios were not significantly altered compared to controls, neither were the phasic E and I charges. The main reason for the phasic I charge being unchanged while detected sIPSC amplitudes being reduced could be that the former was actually incorporating very small currents that remained undetected with traditional thresholding. Inclusion of these events may have weakened the effect of large sIPSCs on reducing the overall sIPSC amplitudes in AD mice. Thus, the unchanged phasic E and I charges resulted in an unchanged E/I ratio. It is also possible that there were compensatory mechanisms taking place in the AD brain to counterbalance the outcomes of decreased inhibition. This possibility will require further investigation. The large variance among different animals and cells when measuring the E/I ratios with our method has to be noted. This could be a result of us choosing multiple segments over long recording durations and the dynamic nature of the E/I balance in the brain. Our E and I recordings were done at different time points at distinct holding voltages. Although we have corrected for the different driving forces, other conditions might be varied when recording E and I. Future analysis using this method should measure E and I at an intermediate voltage (between E_GABA_ and E_Glutamate_) at the same time to control these variations.

Through objective amplitude analysis on the sPSCs, we were able to examine the changes in small and large currents separately. Results revealed that the reduction in the overall average amplitude of the sIPSCs in *App^NL-F^* mice was caused by a decrease in the average amplitude of the large sIPSCs but not the small ones. Moreover, large sIPSCs had significantly faster rates of rise than small ones, and the high RR of large sIPSCs was reduced in *App^NL-F^* mice. These alterations are consistent with a possible PV IN dysfunction in the *App^NL-F^* mice, because PV INs target α1 subunit-containing GABA_A_ receptors on the somatic and perisomatic compartments of their postsynaptic targets, thus generating fast-rising sIPSCs with large amplitudes (Thomson et al., 2000; Nyíri et al., 2001; Klausberger et al., 2002; Freund and Katona, 2007). Altered PV IN inhibition on pyramidal cells is closely linked to abnormal network oscillations and cognitive functions. Network hyperexcitability and epileptiform events have been observed in most mouse models of AD (Palop et al., 2007, 2011; Busche et al., 2008; Palop and Mucke, 2009; Harris et al., 2010; Roberson et al., 2011; Yan et al., 2012; Busche and Konnerth, 2016) and in AD patients using high resolution recordings (Lam et al., 2017). Hypersynchronous network activities have been shown to emerge during reduced gamma oscillation, which is a rhythm that depends on the activities of fast-spiking PV INs and contributes to cognitive functions (Cardin et al., 2009; Sohal et al., 2009; Buzsáki and Wang, 2012; Sohal, 2012; Cho et al., 2015). Specifically, PV IN defects due to a reduced expression of voltage-gated sodium channels Nav1.1 were found in hAPPJ20 mice, and reversing the Nav1.1 reduction by Nav1.1-BAC bacterial rescued PV IN synaptic currents and gamma oscillations and reduced network hypersynchrony and memory deficits in these mice (Verret et al., 2012). A recent study showed that there was no change in the overall Nav1.1 level in either APP-overexpressing or *App^NL-F^* mice brain homogenates, suggesting that Nav1.1 downregulation may be a phenotype unique to hAPPJ20 mice (Saito et al., 2016). However, the fraction of PV IN in brain homogenates, depending on the brain area, may be too small to detect changes in this specific population of interneurons. Therefore, a brain region specific reduction of PV IN Nav1.1 may still be present in *App^NL-F^* mice. Our results based on rigorous sIPSC analyses are consistent with this possibility. Compared to existing hAPP mouse lines widely used to study cerebral Aβ amyloidosis as well as synaptic, network and behavioral dysfunctions (Götz and Ittner, 2008; LaFerla and Green, 2012), the *App^NL-F^* mouse model we used was generated by a gene knock-in approach (Nilsson et al., 2014; Saito et al., 2014) with no hAPP overexpression. However, we have not identified alterations in excitation and inhibition in the cortex in hAPP-overexpressing models, and further studies in those models should be done using the methods described here to investigate the role of hAPP overexpression in E/I balance. Also, studies on the functioning of PV INs in the *App^NL-F^* model are needed, and research on network activities and related cognitive functions could reveal new phenotypes beyond those observed in the hAPP mice.

Studying abnormal synaptic transmission and related aberrant network activities and cognitive deficits in mouse models of AD have valuable translational implications for human AD research. Epilepsy with clinical seizures are particularly more prevalent in sporadic and familial AD patients at younger ages, while overt seizures are not reported in most AD cases (Scarmeas et al., 2009). Therefore, it remains difficult to study potential epileptiform activities in patients with conventional EEG electrodes because the abberant activity takes place in deep temporal structures. A recent study using foramen ovale electrodes positioned adjacent to the mesial temporal lobe revealed clinically silent hippocampal seizures and epileptiform spikes during sleep, a period especially important for memory consolidation (Lam et al., 2017). In urethane-anesthetized *App^NL-F^* mice, abnormal gamma oscillations were observed in the entorhinal cortex, including reduced theta-γ coupling and impaired phase-locking of layer 2/3 pyramidal cell spiking activities (Nakazono et al., 2017). Since PV INs are essential for generating gamma oscillations and for suppressing hypersynchronous network activity, an impairment in PV IN function shared among different mouse models of AD and present during the initial stages of the disease may have highly relevant clinical and therapeutic consequences. The development of specific Na^+^ channel modulators is an obvious start in this direction (Anderson et al., 2017; Frederiksen et al., 2017).

## Synthesis

Reviewing Editor: Lorna Role, Stony Brook Decisions are customarily a result of the Reviewing Editor and the peer reviewers coming together and discussing their recommendations until a consensus is reached. When revisions are invited, a fact-based synthesis statement explaining their decision and outlining what is needed to prepare a revision will be listed below. The following reviewer(s) agreed to reveal their identity: Grace Stutzmann, Geoffrey Murphy. Note: If this manuscript was transferred from JNeurosci and a decision was made to accept the manuscript without peer review, a brief statement to this effect will instead be what is listed below.

This is a moderately revised manuscript which has identified a reduction in large sIPSCs and their RR, in the parietal cortex of 18-20 month old APP KI mouse created by Saido et al. The authors demonstrate a modest imbalance between excitatory and inhibitory synaptic transmission in parietal cortex layer 2/3 pyramidal cells in this AppNL-F KI mouse model of Alzheimer's disease (AD). Specifically, the study reveals that spontaneous inhibitory postsynaptic currents of large amplitude are reduced in the AD mice, while the amplitudes of spontaneous excitatory postsynaptic currents are unaltered.

Given the limited description of the AppNL-F KI mice to date, the characterization per se is important. Nevertheless, the reviewers concurred that while interesting, the majority of data from this study are negative, and the source, mechanism, and implications of the single significant difference are unclear.

In fact, the strength of the paper resides in the methodology for sPSCS acquisition and analysis and in the discussion about network properties. Most interesting are the approaches used to quantify the balance between excitatory/inhibitory synaptic inputs. This coupled with the methods for quantification of the “burstiness” and “memory” aspects of spontaneous postsynaptic currents represent a significant step forward in the field of synaptic physiology.

The reviewers also noted that although the manuscript discusses the advantages of the KI model there was no explicit comparison to conventional transgenic/overexpressing APP mouse models. As such it is unclear if there are distinct advantages to using this model for examining cortical network properties.

Overall, the reviewers concluded that the focus of this paper is stronger from a methods and interpretation perspective, than in terms of the modest findings reported. As such, the reviewers recommend that the presentation is restructured so that the manuscript (starting with rewriting the abstract) better reflects the inherent strengths of the methodological approaches described, rather than in trying to oversell a relatively minor observation.

Minor comments and code related:

1. Please include age of mice in the Methods section.

2. The authors used a custom Igor written procedure to calculate the E/I ratios. They did not provide the code but they do describe what the code accomplished. It would be great if they provided the code or at least stated that they would be willing to share the code (with proper credit).

## Author Response

Synthesis of Reviews:

Computational Neuroscience Model Code Accessibility Comments for Author (Required):

The authors used a custom Igor written procedure to calculate the E/I ratios. They did not provide the code but they do describe what the code accomplished. It would be great if they provided the code or at least stated that they would be willing to share the code (with proper credit).

A statement about sharing the code upon request is now included in the Methods section. Significance Statement Comments for Author (Required):

The significance statement should emphasize the methodological strengths and contributions of the paper, rather than the observation of a modest change in E/I balance.

The significance statement has been revised accordingly Comments on the Visual Abstract for Author (Required): n/a

Synthesis Statement for Author (Required):

This is a moderately revised manuscript which has identified a reduction in large sIPSCs and their RR, in the parietal cortex of 18-20 month old APP KI mouse created by Saido et al. The authors demonstrate a modest imbalance between excitatory and inhibitory synaptic transmission in parietal cortex layer 2/3 pyramidal cells in this AppNL-F KI mouse model of Alzheimer's disease (AD). Specifically, the study reveals that spontaneous inhibitory postsynaptic currents of large amplitude are reduced in the AD mice, while the amplitudes of spontaneous excitatory postsynaptic currents are unaltered.

Given the limited description of the AppNL-F KI mice to date, the characterization per se is important. Nevertheless, the reviewers concurred that while interesting, the majority of data from this study are negative, and the source, mechanism, and implications of the single significant difference are unclear.

The reduced large amplitude fast rise rate sIPSCs are consistent with a dysfunction of PV+ interneurons in the parietal cortex, but identifying the precise mechanism(s) for the dysfunction were outside the scope of our investigation. Nevertheless, we have performed a thorough analysis of the spontaneous synaptic events, and have shown that compensatory mechanisms re-balance the excitation/inhibition in the AD mice. The significance of these, at first sight, subtle alterations on the development of the Ab plaques remains to be determined.

In fact, the strength of the paper resides in the methodology for sPSCS acquisition and analysis and in the discussion about network properties. Most interesting are the approaches used to quantify the balance between excitatory/inhibitory synaptic inputs. This coupled with the methods for quantification

of the “burstiness” and “memory” aspects of spontaneous postsynaptic currents represent a significant step forward in the field of synaptic physiology.

We thank the reviewers for highlighting the strengths of our study. We have made revisions according to the suggestions.

The reviewers also noted that although the manuscript discusses the advantages of the KI model there was no explicit comparison to conventional transgenic/overexpressing APP mouse models. As such it is unclear if there are distinct advantages to using this model for examining cortical network properties.

As outlined in the ms and references therein, many of the APP overexpressing transgenic mouse models of AD are considered to produce artifacts due to the ectopic expression of mutant human APP on top of the mouse APP. Therefore, our intent was not to compare the APP(NL-F) mice to these previous

models, but to build a database for the synaptic function in these mice for later comparison to other

second and third generation mouse models of AD.

Overall, the reviewers concluded that the focus of this paper is stronger from a methods and interpretation perspective, than in terms of the modest findings reported. As such, the reviewers recommend that the presentation is restructured so that the manuscript (starting with rewriting the abstract) better reflects the inherent strengths of the methodological approaches described, rather than in trying to oversell a relatively minor observation.

We have revised the ms accordingly. Minor comments and code related:

1. Please include age of mice in the Methods section.

The age and sex of the mice are now included in the Methods section

2. The authors used a custom Igor written procedure to calculate the E/I ratios. They did not provide the code but they do describe what the code accomplished. It would be great if they provided the code or at least stated that they would be willing to share the code (with proper credit).

A statement about sharing the code upon request is now included in the Methods section.
